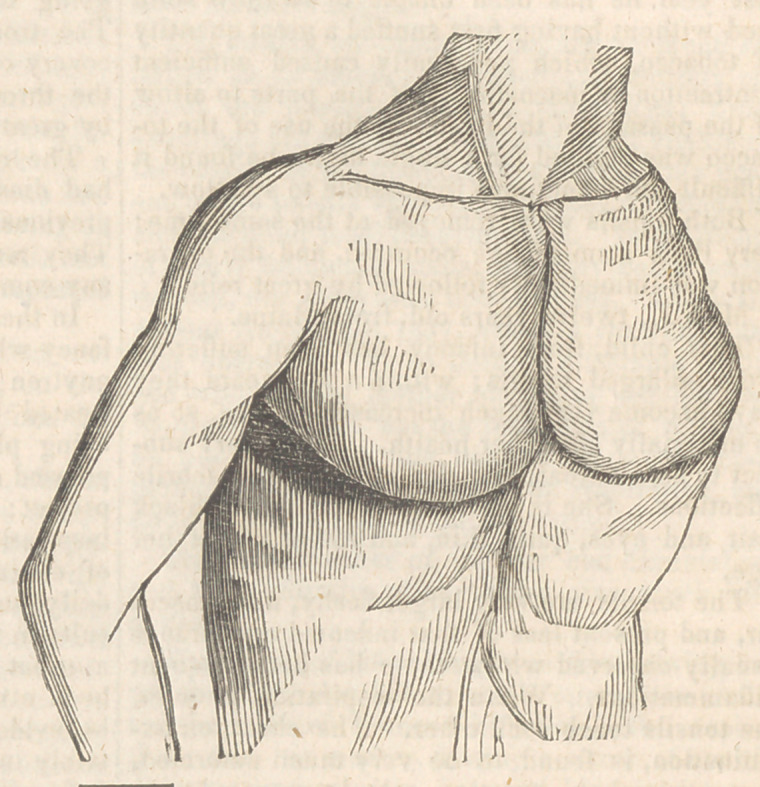# Remarks on Enlargement of the Tonsils, Attended by Certain Deformities of the Chest

**Published:** 1839-05-18

**Authors:** J. Mason Warren


### MEDICAL EXAMINER.

DEVOTED TO MEDICINE, SURGERY, AND THE COLLATERAL SCIENCES.

No. 20.] PHILADELPHIA, SATURDAY, MAY 18, 1839. [Vol. II.

*Remarks on Enlargement of the Tonsils, attended by certain Deformities of the Chest.* By J. Mason Warren, M. D.

[Read before the Boston Society for Medical Improvement.]

Within a few years the attention of the medical public has been much called to a very common affection, most frequently observed in children, viz. an enlarged state of the tonsils. The great constitutional derangement which often arises from the existence of an obstruction in the throat to the free passage of the air and of the aliments, causes the disease to be of a more serious character than would, at first, be apprehended, and makes it desirable that some certain method should be adopted for its relief.

Formerly, after an ineffectual resort to caustics, blisters, and various other internal and external applications, excision by the bistoury was resorted to; this becomes a very serious operation, from the frequent and alarming hæmorrhage which often follows, and is, therefore, submitted to with great reluctance by the patient, and approached with some diffidence by the surgeon. The ligature is so tedious and painful in its application, as to be allowed, at the present day, to fall entirely into disuse.

The object of this paper is to point out, by the exhibition of a number of cases, the certainty and ease with which the operation may be performed with the present improved instrument, and the great relief always experienced by the removal of these organs when in an enlarged state.

In the remarks which will be offered on certain deformities of the chest which complicate this disease, nothing original is intended; they may serve, however, to draw the attention of the society to the relation which exists between the enlargement of the tonsils and this affection, and the results of the case will lead us to suspect that the symptoms which have been ascribed to this deformity, may be more referrible to the obstruction which exists in the throat.

In 1827, M. Dupuytren published a paper on the lateral depression of the parietes of the chest, consisting of a depression more or less great of the ribs on each side, and a proportionate protrusion of the sternum in front, accompanied by some antero-posterior curvature of the vertebral column. A portion of these cases occurred in children of a scrofulous habit, and were invariably accompanied by an enlargement of the tonsils.

The symptoms described by M. Dupuytren as attending this disease, were habitual shortness of breath, and difficulty of enunciation. With infants there was great difficulty in taking the breast, the child being threatened with suffocation whenever the nipple was detained for any length of time in the mouth. During sleep, the mouth was kept habitually open, and the respiration accompanied by great noise, and frequently interrupted by frightful dreams and cries. "These symptoms," says M. Dupuytren, "may be increased so as to prevent the development of the vital functions, and cause death in the earliest period of life. When these difficulties do not induce death immediately, they may destroy life at a later period, either in preventing the child from taking the breast, or in so altering the nutrition as to prevent the development of the strength of the different organs; in this case death does not at once take place, but the child lives in a miserable state of feebleness and emaciation, which deprives him of the greater part of his faculties."

In 1827, shortly after the publication of this paper, Mr. Coalson, of London, published some cases in confirmation of those given by Dupuytren, adding, also, three cases of his own, of a deformity of the chest, different from that before described. "The external appearances of the chest," says Mr. Coalson, "in this second kind of deformity, are directly the reverse of those which we have just been considering. The sternum is hollow or concave anteriorly, the sides of the chest are very prominent, and the spinal column but slightly, if in any degree, altered from its natural shape; this is not so frequently congenital as the former kind, but frequently occurs in persons of a weak habit, who are narrow-chested, and stoop a great deal. The constitutional symptoms are very much the same as those attendant on the other kind of deformity." On the three cases appended to the paper of Mr. Coalson, and three of the four cases of M. Dupuytren, enlargement of the tonsils existed; but in none of them does it appear that removal of these organs was practised, although it is stated that in one or two of them the tonsils were so large as nearly to fill up the posterior part of the fauces, so that we are not enabled to judge of what would have been the change effected on the symptoms referred to the chest, had this operation been performed.

Within the last two years twenty cases have occurred in our practice, in which it was thought necessary that an operation for the removal of the tonsils should be practised: in nineteen of these cases the operation was successfully performed; in one case the tonsils projected so little into the throat, as to make it impossible to seize them with the instrument. The operation was temporarily deferred. Of these twenty cases, fifteen were children, or less than twelve years of age.

Of the fifteen children, eleven had more or less deformity of the chest, consisting, in the greater number, of a projection of the cartilages of the ribs forwards, with a considerable excavation of the sternum. In these patients very little curvature could be detected in the spinal column.

In the five adults, no alteration of the parietes of the chest was perceptible.

The symptoms occurring in these patients were as follows:

In every one of them was more or less difficulty in respiration, in many cases the noise being so great during sleep as to make it impossible for any person to sleep in the same room; the sleep was often disturbed by frightful dreams.

In many of the patients there was great difficulty of swallowing, liquid food being often regurgitated into the nostrils; in one case, no solid food could be taken without the previous use of a powerful astringent. About half the cases were attended with severe constitutional symptoms.

In one case entire deafness was present. Some of the patients were liable to periodical attacks of fever; in one case, a child, five years of age, returning, latterly, as often as once a fortnight, and lasting three or four days. Eight of the fifteen children showed more or less marks of a scrofulous habit. Eighteen of the patients had both tonsils removed; the other patient being so much relieved by the removal of one tonsil, that it was unnecessary to have the operation repeated on the other side.

In about half the patients this operation was performed on both sides the same day; in the others a week was allowed to elapse before the other tonsil was removed.

In eighteen out of the nineteen cases, almost immediate relief was afforded to all the symptoms; in the other case, no great relief was apparent, and this seemed to be attributable to the particular shape of these organs, the base being quite broad, and extending some distance down the throat, about half of each tonsil was removed. At the end of a short period, an appearance was presented as if they had been again regenerated; this arose from the upper and lower portions rising or curling up, as it were, after the apex had been removed. At the end of two years, this patient submitted to a second operation, followed by much relief, and is now in a fair way of recovery.

The operation, as performed by the present improved instrument, is instantaneous,—not attended with much pain,—in no case was there any considerable hæmorrhage,—usually nothing more than a few mouthfuls of blood are discharged. The patients are able to return home and resume their ordinary occupations, as if nothing uncommon had occurred, a slight soreness only being experienced for the few following days.

We now proceed to offer one or two cases illustrative of the different symptoms of the disease, and may first select one which will present most of the symptoms occurring in the course of it.

W., of Newton, Mass., five years of age, November, 1836. For the last two years this child has been troubled by an enlargement of the tonsils, first manifested by a swelling which appeared on the outside of the throat, and supposed by the parents, at the time, to be mumps. As the disease increased, the patient gradually lost his his flesh and strength, and was subject to frequent sore throat, attended by febrile attacks, these latterly occurring as often as once in a fortnight, and lasting two or three days; his breathing at night was very difficult, and accompanied with much noise. The ear of one side was inflamed, attended with a purulent discharge; he was very sensitive to any loud musical sounds. He is small of his age, thin, of an irritable disposition. The chest, on examination, is found to be much deformed, presenting that appearance called excavated sternum, it being very much depressed in its centre, and the ribs at their union with the cartilages elevated so as to form with them an acute angle.

The tonsils, on examination, are so much enlarged as to touch each other, and entirely obstruct the posterior part of the fauces; these swellings are distinctly felt, and even visible on the outside of the throat, at the angle of the jaw; one of the tonsils was removed, and afforded immediate relief to all the symptoms. In the month of April following, some difficulty being experienced, the other was also excised. I saw the patient August 3d, 1837, nearly a year after the first operation. From being a miserable child, and who, as his mother stated, to use her own words, "she had not the least idea of raising," he has become a fine healthy boy,—has been perfectly free from difficulty of respiration, and no febrile attack since the operation.

The sensitiveness of the ear had diminished, and the deformity of the chest was much less obvious.

The object of his calling, was from having experienced the day before some oppression at the stomach, which induces difficulty in the respiration; and his mother, fearing a return of his old disease, immediately brought him into town. The symptoms were explained, by his having passed a fortnight absent from home, where he had been allowed rather too much freedom in his diet.

The following is the case of a person of a more advanced age, in which deafness was produced by the disease:

B., aged 18—November, 1836.

For two or three years has been subject to frequent attacks of sore throat; for three months has had a purulent discharge from the right ear; is now quite deaf in both ears, so as to require to be spoken to in a very loud voice. It is for this deafness that he applies for advice.

On examination of the ears by the speculum, the tympanum on both sides was found to be in a perfectly sound state; on the side from which the discharge appears, the lining membrane of the ear is reddened, and covered by a purulent deposit. The patient bears all the marks of a scrofulous constitution. The tonsils are found to be very much enlarged, attended with considerable redness of the back part of the fauces.

Astringent remedies being tried for a fortnight without effect, both tonsils were removed. On the following day he began to hear better; on the second day his hearing was perfectly restored, and sounds became even so acute as to be painful.

In a day or two the deafness returned, and lasted a week; he then recovered his hearing, and has remained perfectly well since. I have seen him lately, more than two years having elapsed since the operation; and he has experienced no return of his difficulty.

*January*, 1838.—A gentleman, twenty years of age, from the Western country, called on me with the tonsils greatly enlarged; he had been troubled with this affection for five years, and has tried many applications without effect. For the last year he has been unable to swallow solid food without having first snuffed a great quantity of tobacco, which apparently caused sufficient contraction or insensibility of the parts to allow of the passage of the food. If the use of the tobacco was omitted for a single night, he found it difficult and sometimes impossible to swallow.

Both tonsils were removed at the same time; very little hæmorrhage occurred, and the operation was immediately followed by great relief.

Miss J., twelve years old, from Maine.

This child, from infancy, has been suffering from enlarged tonsils; within a few years they have become very much increased in size, so as to materially affect her health. She is very subject to sore throat, attended with severe febrile affections. She is of a dark complexion, black hair and eyes, quite thin, and rather tall of her age.

The tonsils are very large, fleshy, and vascular, and present less of that indented appearance usually observed where there has been frequent inflammations. When the respiration is quiet, the tonsils touch each other. The chest, on examination, is found to be very much deformed, presenting that alteration, called excavated sternum, in its most exaggerated form; the hollow lining almost large enough to contain a small orange: this deformity has been, for many years, observed by her parents.

The breathing at night is very difficult and noisy; she is subject to attacks of deafness, and at present does not hear unless addressed in a loud voice.

The right tonsil was removed on the 12th, and the patient at once relieved by it; five days afterwards the other tonsil was removed, leaving the throat perfectly free. On the 25th I saw the patient, and the mother informed me that all the previous bad symptoms were removed—that the child has quite recovered her health. The difficulty of breathing is relieved, and her hearing returned; a cutaneous eruption which had long troubled her, has disappeared.

To these cases might be added one or two in which these organs were removed while the patient was labouring under an attack of severe tonsilitis. In one case the symptoms were immediately removed by the operation; in another, inflammation had extended to the adjacent parts, and an abscess formed, as is often seen in this disease. The affection, however, was much shortened in duration, lasting four days, instead of fourteen, as had been usual with this patient, who was liable to attacks every winter. The operation was repeated, and the other tonsil was removed on a subsequent attack, with the same result.

Some time since I communicated to this society the case of a young child from Maine, who was brought to Boston suffering from a disease of the throat. The parents seemed to be quite unconscious of the cause of its troubles. There was great difficulty of breathing and deglutition. The child had a spoon, the bowl of which it placed almost instinctively in its mouth when going to sleep; its health was very miserable. The trouble was entirely explained by the discovery of the enlarged tonsils quite obstructing the throat. The removal of them was followed by great relief.

The mother informed me that another child had died with the same symptoms a few years previous, the cause of its illness being unknown. They resided in an obscure spot, distant from any competent medical advice.

In these cases of the disease occurring in infancy where deformity of the chest exists, Dupuytren advises that this affection should be treated in the following manner:—The child being placed in the lap of its nurse, the hand is pressed on that part of the sternum or ribs which project; a strong pressure is then made during inspiration, and removed during the movements of expiration. This repeated for many times daily, and continued for a long period, finally results in the disappearance of the deformity, or in a great improvement of appearance. As has been attempted to show above, however, it will be evident to all that the symptoms arise, certainly in the great number of cases, not from the deformity, but from the obstruction in the throat to the free passage of air.

The instrument* used in these operations, has usually been the guillotine instrument, as described by Dr. Warren in his work on Tumours, being somewhat similar to that of Dr. Physick; it is, however, without the steel moveable needle, used to fix the tonsil and prevent it from falling into the throat, which appears to be useless, as the blade of the instrument drives the lining membrane of the tonsils into its groove, and thus secures it; and even if this were not the case, the mucus which covers the fauces causes the excised part to adhere to the blade, so that there is no danger of its escaping into the throat.

In very young children, where the passage of the fauces is narrower, a more delicate instrument, invented by Dr. Fahnestock, of Pennsylvania, is, perhaps, preferable.

From a review of the above cases, we shall

* Invented by Caleb Eddy, Esq., of Boston.

find that many of the children are of a scrofulous constitution,—that the enlargement of the tonsils causes great local trouble, attended with considerable constitutional disturbance,—that the patient is much more liable to inflammatory attacks of the throat, than in cases where this enlargement does not exist,—and that they are less liable, *after the operation, to these attacks.*

In about half of all the cases, and in about two-thirds of the cases of children, deformity of the chest exists. Whether this depends on the general constitutional habit of the patient, or is induced by the obstruction in the throat to the free passage of air, the accounts received from parents as to the exact time when either affection was first observed, are not sufficiently accurate to permit us to determine; it is certain, however, that this deformity does not increase, but rather diminishes after the removal of the tonsils. The operation is a simple one, attended with no danger, and almost always affords immediate relief to the symptoms.

To this paper might be added an account of the appearance of the diseased tonsil when removed, and various other circumstances relating to the operation; but as the limits of the paper will not allow of a farther extension, this for the present must be omitted.

## Figures and Tables

**Figure f1:**
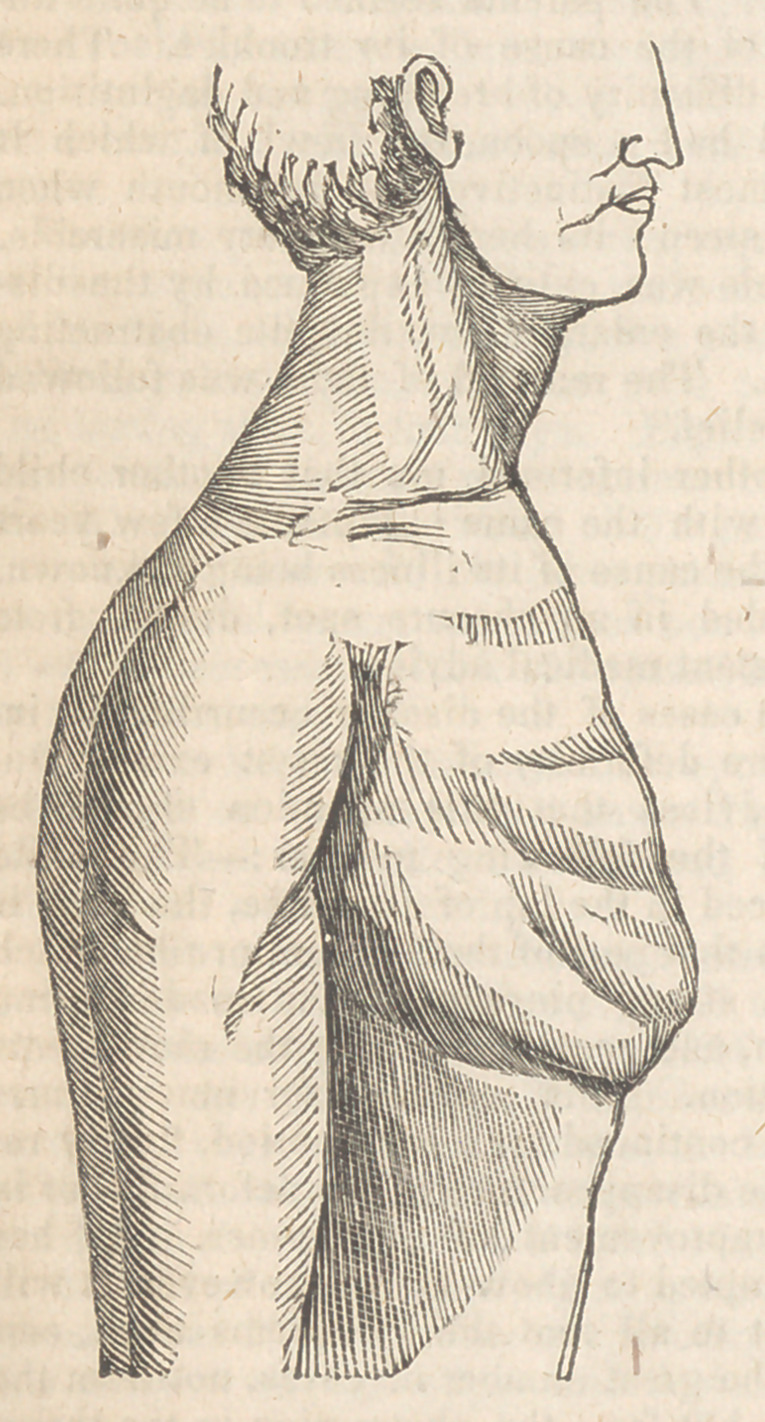


**Figure f2:**